# Dietary intake as a predictor for all-cause mortality in hemodialysis subjects (NUGE-HD study)

**DOI:** 10.1371/journal.pone.0226568

**Published:** 2019-12-17

**Authors:** Karla Pereira Balbino, Leidjaira Lopes Juvanhol, Andreza de Paula Santos Epifânio, Luciane Domingos Marota, Josefina Bressan, Helen Hermana Miranda Hermsdorff

**Affiliations:** 1 Department of Nutrition and Health. Universidade Federal de Viçosa, Viçosa, Minas Gerais, Brazil; 2 Division of Nephrology, São João Batista Hospital, Viçosa, Minas Gerais, Brazil; Purdue University, UNITED STATES

## Abstract

This study aimed to identify the factors capable of mortality prediction in patients on hemodialysis, using a prospective cohort with three years of follow-up. We hypothesized that lack of clinical-metabolic control, impairment of nutritional status, and inadequate food consumption are risk factors for mortality in this population. This is a longitudinal study on a non-probabilistic sample of 85 adults and elderly patients undergoing hemodialysis, aged ≥ 18 years (66.0% male, 61.6±13.7 years). Data on anthropometric, biomarkers, body composition and food intake were obtained. Predictors of mortality were evaluated using Cox regression analysis. During the three years follow-up, 16 patients (18.8%) died. We observed that age (HR = 1.319, CI 95% = 1.131–1.538), calcium-phosphorus product (HR = 1.114, CI 95% = 1.031–1.205), ferritin (HR = 1.001, CI 95% = 1.001–1.002), nitric oxide (HR = 1.082, CI 95% = 1.006–1.164), and vitamin C intake (HR = 1.005, CI 95% = 1.001–1.009) were positively associated with mortality. Serum iron (HR = 0.717, CI 95% = 0.567–0.907), triceps skinfold thickness (HR = 0.704, CI 95% = 0.519–0.954), lean mass (HR = 0.863, CI 95% = 0.787–0.945), and the ratio of dietary monounsaturated/polyunsaturated fat (HR = 0.022, CI 95% = 0.001–0.549) were independent negative predictors of mortality. Our results suggest that dietary intake is also a predictor of mortality in patients on hemodialysis, besides nutritional status, body composition, oxidative stress, inflammation, and bone metabolism, indicating the importance of evaluation of these factors altogether for better prognosis.

## Introduction

The prevalence and incidence of end-stage kidney disease are increasing worldwide. About 70% of people who progress to this stage of disease (approximately three million people worldwide) are treated with hemodialysis (HD) [1,2]. Although HD is effective in increasing survival rates, the mortality among patients undergoing HD is still high [3,4]. Cardiovascular disease (CVD) is the leading cause of death in patients on HD [5], associated with a higher prevalence of traditional risk factors, such as hypertension, diabetes mellitus (DM), dyslipidemia, smoking, and old age. In addition, water overload, hyperphosphatemia, elevated calcium-phosphorus product, anemia, left ventricular hypertrophy, inflammation, oxidative stress, endothelial dysfunction, insulin resistance, hyperhomocysteinemia, and elevated serum lipoprotein (a) concentration are all prevalent conditions which increase the morbidity and mortality in patients on HD [6,7].

In addition to CVD, studies have highlighted the importance of non-cardiovascular causes of death in these patients, which was poorly evaluated until then [8]. In this context, mineral and bone disorders are common occurrences in chronic kidney disease and are mainly due to abnormalities in serum levels of calcium, phosphorus, and parathyroid hormone (PTH). Changes in mineral metabolism have been shown to be associated with the morbidity and mortality of patients on HD [9,10].

Finally, systemic inflammation and malnutrition also contribute directly to increased mortality and hospitalizations and are mutually dependent [11]. Malnutrition is a result of inadequate food intake caused by anorexia, underlying diseases, psychosocial conditions, aging, or by chronic inflammation itself [12]. In fact, most patients on HD consume nutrients, especially caloric-protein, in amounts below the recommendations, throughout the treatment [13,14]. However, the impact of dietary intake on the mortality of these patients is still little studied.

We hypothesized that lack of clinical-metabolic control, impairment of nutritional status, and inadequate food consumption are risk factors for mortality in this population. Therefore, the aim of the present study was to identify the predictors of all-cause mortality in patients on HD after three years of follow-up. In this study, we were able to identify these factors and noted their importance in the prognosis and survival improvement of these patients.

## Materials and methods

### Studied population

This is a longitudinal study from the cohort Nutrition and Genetics on Hemodialysis outcomes (NUGE-HD) with 85 patients on HD treatment (56 men, 29 women, mean age: 61.6 years, SD = 13.7 years, range 19–86 years), treated at a single dialysis center. Patients underwent three weekly sessions of HD, lasting 3–4 hours, with blood flow greater than 250 mL/min and dialysate flow of 500 mL/min.

The inclusion criteria for the study were 18 years of age or older and a treatment time in HD of more than one month, whereas the criteria for non-inclusion were auditory deficiency (informed by sector employees), newly implanted catheters, instability hemodynamics (according to the service protocol), and inability to stand for anthropometric measurements. All individuals undergoing hemodialysis who presented these adequate criteria were invited to participate in the study. After explanation of study’s objectives and procedures of data collection, those individuals who wished to participate read and signed a written informed consent in accordance with the principles of Declaration of Helsinki. The study was approved by the Human Research Ethics Committee of the Universidade Federal de Viçosa (N° 701.796/2014).

### Follow-up

The survival time was measured in days, from the date of the first interview, which occurred between September and December of 2014, until death or end of follow-up (October 2017), whichever occurred first. To obtain information on the outcome, such as death dates and the basic cause of death, medical records were consulted in the HD service in order to verify if the patients whose data was collected at the beginning of the study were alive or not. These data were then checked in the Mortality Information System (http://datasus.saude.gov.br/sistemas-e-aplicativos/eventos-v/sim-sistema-de-informacoes-de-mortalidade), in which information on the basic cause of death (ICD-10) was also stored. Patients who underwent renal transplantation were censored at the time of transplantation and all patients alive at the end of follow-up were censored at the time of last contact with the researchers.

### Sociodemographic variables

The patients were interviewed using a semi-structured sociodemographic and health questionnaire (Questionnaire in S1 File), and information on clinical history, scholarity, consumption of alcoholic beverages, and smoking were obtained. The medical records of each patient were carefully reviewed and data related to underlying kidney disease, cardiovascular history, or other comorbidities were collected. Patients with current smoking habits were considered smokers.

### Biomarkers assessment

Data on serum concentrations of hemoglobin, hematocrit, erythrocytes, creatinine, pre-dialysis urea, albumin, calcium, phosphorus, PTH, potassium, iron, ferritin, total iron-binding capacity (TIBC), C-reactive protein (CRP), total cholesterol, and triglycerides were obtained by consulting medical records and determined using routine laboratory techniques. To measure all these markers, non-fasting blood samples were collected before initiation of dialysis. Serum calcium corrected for albumin levels was calculated according to the formula: corrected calcium = total serum calcium + [(4-serum albumin) x 0.8]. Kt/V urea was calculated using the equation proposed by Daugirdas II [15]. Values of Kt/V urea > 1.2 were considered indicative of HD efficacy [15]. For other variables, the alterations were evaluated according to reference values (Table 1).

**Table 1:** Reference values of biochemical, anthropometric and body composition variables of patients on hemodialysis.

	Reference value
Biochemical Variables	
Calcium, mmol/L [16]	2.10–2.37
Phosphorus, mmol/L [16]	1.13–1.78
Calcium-phosphorus product, mmol^2^/L^2^ [16]	< 4.4
Parathormone, pmol/L [16]	16.5–33.0
Potassium, mmol/L [17]	≤ 5.5
Hemoglobin, g/L [18]	110.0–130.0
Iron, μmol/L [18]	9.0–26.9
Ferritin, pmol/L [18]	449.4–1123.5
Saturation of transferrin, % [18]	20.0–40.0
Albumin, g/L [19]	>38.0 a 40.0
Total cholesterol, mmol/L [20]	< 5.18
Triglycerides, mmol/L [21]	< 1.70
C-reactive protein, mg/L [22]	≤ 3.0
**Body Mass Index** [25,26]	
Underweight	Adult: < 18.5 kg/m^2^
	Elderly: < 22 kg/m^2^
Normal-weight	Adult: 18.5–24.9 kg/m^2^
	Elderly: 22–27 kg/m^2^
Excess body weight	Adult: ≥ 25 kg/m^2^
	Elderly: > 27 kg/m^2^
**Waist Circumference** [27]	
High risk for metabolic complications	Woman: ≥ 80 cm
	Men: ≥ 94 cm
Very high risk for metabolic complications	Woman: ≥ 88 cm
	Men: ≥ 102 cm
**% Body Fat** [28,29]	
Fat shortage	Woman: • 18–39 years: < 21% • 40–59 years: < 23% • 60–99 years: < 34%
	Men: • 18–39 years: < 8% • 40–59 years: < 11% • 60–99 years: < 13%
Excess fat	Woman: • 18–39 years: > 33% • 40–59 years: > 34% • 60–99 years: < 36%
	Men: • 18–39 years: > 20% • 40–59 years: > 22% • 60–99 years: > 25%

### Determination of nitric oxide

On the day of routine biochemical exams, the serum was aliquoted and stored at -80°C for further analysis of nitric oxide (NO) using standardized protocols as previously described [23].

### Anthropometric and body composition assessment

The anthropometric and body composition assessment was performed approximately 30 minutes after the end of the HD session. Body weight (BW, kg), height (cm), mid-arm circumference (MAC, cm), triceps skinfold thicknesses (TSF, mm) and waist circumference (WC) were measured using standard techniques, as previously described [24]. From the MAC and TSF measurements were calculated mid-arm muscle circumference (MAMC), corrected mid-arm muscle circumference (CMAMC) and mid-arm fat area (MAFA), according to the formulas below. Muscle mass and fat were determined by electrical bioimpedance (BIA; TANITA^®^, model BC150, Tokyo, Japan). The cutoff values used to classify patients according to body mass index (BMI), WC, and percentage of body fat are presented in Table 1.

MAMC (cm) = MAC (cm)– π x [TSF (mm) ÷ 10]

π = 3.1416

- Men:

$$CMAMC\;(cm2)=\frac{[MAC\;(cm)-\pi \;X\;TSF\;(cm)]2}{4\pi }-10$$

- Women:

$$CMAMC\;(cm2)=\frac{[MAC\;(cm)-\pi \;X\;TSF\;(cm)]2}{4\pi }-6.5$$

$$MAFA\;(cm2)=\frac{MAMC\;(cm)X[TSF\;(mm)/10]}{2}-\frac{\pi [TSF\;(mm)/10]2}{4}$$

### Food consumption

To estimate food consumption, we adapted a food frequency questionnaire (FFQ) for renal patients [30]. According to the Food Guide for the Brazilian Population [31], food portions of food groups such as cereals, tubers and roots; fruits and vegetables; beans and vegetable foods rich in protein; milk and dairy products; meat and eggs; fats, sugars and salt; beverages, and oilseeds were analyzed. During routine HD sessions in the dialysis unit, the FFQ was used by trained researchers to interview patients. For patients with any sign of cognitive impairment, the responses were confirmed with care takers. During the interview, a photographic album [32] with similar portions was used, so that the interviewee chose the categories of food portions from the album, corresponding to the habitual intake.

To calculate dietary intake, consumption of each food item was converted to grams per day and the daily consumption of each nutrient was calculated, according to the nutritional composition of Brazilian food tables [33,34], using a Microsoft^®^ Excel spreadsheet, especially designed for this. In this study, we evaluated caloric intake (kcal/kg BW), carbohydrates (g/kg BW), protein (g/kg BW), total lipids (g/kg BW) and fatty acids profile (% of caloric intake—CI), total cholesterol (mg), calcium (mg), phosphorus (mg), potassium (mg), sodium (mg), iron (mg), vitamin E (IU), and vitamin C (mg) quantitatively. Ratio between monounsaturated (g) and polyunsaturated (g; MUFA/PUFA), monounsaturated (g) and saturated (g; MUFA/SFA) and polyunsaturated and saturated (g; PUFA/SFA) dietary fatty acids were also calculated. All the nutrients evaluated in the present study were adjusted for daily caloric intake by the residual method [35], before statistical analysis, to minimize the effect of caloric intake on the relationship of consumption variables with all-cause mortality outcome.

### Statistical analyses

Data are presented as mean ± standard deviation (SD) or median (interquartile range), depending on the distribution of variables, as assessed by the Shapiro-Wilk test. Differences between survivors and non-survivors were analyzed using Student's t-test or Mann-Whitney U test for quantitative variables. The chi-square or exact Fischer test was performed to verify the differences between the two groups, regarding categorical variables.

Cox proportional hazard analysis was used to evaluate the independent predictors of mortality. The independent variables to be tested in the Cox model were defined from the literature [36,37]. The forward variable selection method was used. The variable with the lowest *p* value in the crude analysis was initially introduced in the model and this procedure was repeated for all the other independent variables. All variables with *p* values <0.05 remained in the final model.

Hazard Ratios (HR) with 95% confidence intervals (CI) were calculated. The overall fit of the model was evaluated by the explanatory power (R^2^ of the chosen model/R^2^ of the saturated model). All analyses were performed using the software Statistical Package for Social Sciences (SPSS 20.0 for Windows; Chicago, IL, USA) and R 3.4.4, and the level of significance (α) adopted was 0.05.

## Results

There was no loss of participants during follow-up. At the end of three years, 18 patients died (18.8%), with an average follow-up time of 995 days. Eight patients (50%) died due to cardiovascular causes, including ischemic cardiomyopathy, cerebral atherosclerosis, myocardial acute infarction, and congestive heart failure. Other causes, such as classical dengue fever, chronic respiratory failure, and non-traumatic subdural hemorrhage were responsible for the other deaths.

The demographic, clinical, and lifestyle characteristics of the survivors and non-survivors are shown in Table 2. Patients were on HD therapy for a median time of 3.3 years (1.5–7.0 years) when they were included in this study. The main causes for renal disease were hypertensive nephrosclerosis (n = 35), followed by DM (n = 28), glomerulonephritis (n = 13), and other diseases (n = 9). In addition to systemic arterial hypertension and DM (74.1%), dyslipidemia (68.2%), smoking (15.3%), and alcoholism (11.8%) were also highlighted as cardiovascular risk factors. There were no significant differences in the sociodemographic, clinical, and lifestyle characteristics of survivors and non-survivors (Table 2), except for age, since patients who died were found to be older.

**Table 2:** Sociodemographic, clinical and lifestyle characteristics of survivors and non-survivors in hemodialysis at three years of follow-up.

	Total (n = 85)	Survivors (n = 69)	Non-survivors (n = 16)
Follow-up (days)	1127 (961–1141)	1134 (1122–1142)	632 (402–853)*
**Sociodemographic characteristics**			
Age, years	61 (55–71)	60 (54–67)	77 (67–81)*
Sex, male, n (%)	56 (65.9)	46 (66.7)	10 (62.5)
Schooling, <8 years of study, n (%)	62 (72.9)	52 (75.4)	10 (62.5)
**Clinical characteristics**			
HD time, months	40 (18–84)	36 (18–97)	63 (11–75)
Kt/V	1.5 (1.3–1.8)	1.5 (1.2–1.8)	1.6 (1.3–2.1)
Nitric oxide, μmol/L	4.5 (2.3–15.1)	4.3 (2.3–12.6)	8.5 (2.4–16.3)
**Risk factors**			
Consumption of alcoholic beverage, n (%)	10 (11.8)	9 (13.0)	1 (6.2)
Smoking, n (%)	13 (15.3)	13 (18.8)	0 (0)
Dyslipidemia, n (%)	58 (68.2)	47 (68.1)	11 (68.8)
**Etiology for CKD**			
Hypertensive nephrosclerosis, n (%)	35 (41.2)	28 (40.6)	7 (43.8)
Diabetes mellitus, n (%)	28 (32.9)	24 (34.8)	4 (25.0)
Glomerulonephritis, n (%)	13 (15.3)	11 (15.9)	2 (12.5)
Others, n (%)	9 (10.6)	6 (8.7)	3 (18.8)
**Usual food consumption**			
Caloric intake, kcal/kg BW	42.0 (28.8–56.6)	42.4 (27.5–60.8)	39.0 (30.6–46.8)
Carbohydrate, g/kg BW	6.4 (5.5–7.4)	6.4 (5.4–7.4)	6.4 (5.7–8.4)
Protein, g/kg BW	1.4±0.4	1.4±0.4	1.5±0.5
Lipids, g/kg BW	1.6 (1.3–2.0)	1.6 (1.3–1.9)	1.8 (1.3–2.3)
Cholesterol, mg	261.9 (202.5–327.9)	261.8 (203.5–330.5)	244.0 (191.7–309.7)
Saturated fat, % CI	10.0 (6.8–15.2)	9.8 (6.5–15.2)	11.1 (8.0–16.1)
Monounsaturated fat, % CI	11.5 (7.7–14.8)	10.5 (7.3–15.4)	12.7 (8.9–14.5)
Polyunsaturated fat, % CI	13.1 (8.1–17.8)	11.8 (7.9–17.9)	14.5 (9.0–17.5)
Sodium, mg	1718 (1234–2226)	1764 (1242–2226)	1663 (923–2135)
Calcium, mg	715.3 (539.7–885.7)	717.2 (539.9–885.7)	663.3 (535.0–952.3)
Phosphorus, mg	1124 (923–1350)	1124 (905–1326)	1139 (930–1410)
Potassium, mg	2936±864	2939±900	2926±716
Iron, mg	21.1 (9.2–31.6)	21.4 (7.3–36.1)	20.0 (10.9–24.0)
Vitamin E, UI	6.6 (5.9–7.9)	6.5 (5.7–8.0)	6.7 (6.3–7.2)
Vitamin C,mg	185.4 (127.9–272.6)	185.4 (129.9–262.8)	201.6 (114.6–315.6)

Data are expressed as mean ± SD or median (P25—P75). BW: body weight; CI: caloric intake; CKD: chronic kidney disease; g: grams; HD: hemodialysis; kcal: kilocalories; kg: kilograms; mg: milligrams.

*p <0.001 by the Mann-Whitney U test.

Food intake above the recommended amount was observed for cholesterol (> 200 mg) in 70.2%, saturated fat (> 7% of CI) in 67.9%, polyunsaturated fat (> 10% of CI) in 57.1%, potassium (> 2730 mg) in 61.9% and phosphorus (> 700 mg) in 90.5% of the subjects. The protein intake was below 1.1 g/kg body weight in 16.7% of the participants.

Moreover, no significant difference was observed in the biomarkers, nutritional and body composition data of the study population categorized as survivors and non-survivors (Table 3). In 48.8% of the patients, the serum hemoglobin was below 110.0 g/L, in 27.4%, ferritin was lower than 449.4 pmol/L, and the transferrin saturation rate was less than 20% in 25.0% of them, considering the recommended reference values.

**Table 3:** Biomarkers, nutritional and body composition data of survivors and non-survivors in hemodialysis at three years of follow-up.

	Total (n = 85)	Survivors (n = 69)	Non-survivors (n = 16)
**Metabolic variables**			
Creatinine, μmol/L	716.0±229.8	733.73±203.3	627.6±309.4
Pre-dialysis urea, mmol/L	46.6±12.8	46.8±12.9	46.1±12.9
Calcium, mmol/L	2.3 (2.1–2.4)	2.3 (2.1–2.4)	2.2 (2.1–2.4)
Phosphorus, mmol/L	1.5 (1.3–1.7)	1.5 (1.2–1.7)	1.5 (1.4–1.8)
Ca-P product, mmol^2^/L^2^	3.2 (2.7–4.0)	3.2 (2.7–3.9)	3.5 (2.8–4.4)
Parathormone, pmol/L	270.9 (172.3–593.1)	270.9 (186.8–584.9)	335.2 (138.0–650.3)
Potassium, mmol/L	5.5 (5.0–6.4)	5.5 (5.1–6.4)	5.4 (4.8–6.8)
**Hematological variables**			
Red blood cell, (x 10^9^/L)	4.0 (3.0–4.0)	4.0 (3.0–4.0)	3.5 (3.0–4.0)
Hemoglobin, g/L	111.0 (94.0–121.0)	111.0 (94.0–121.0)	108.0 (90.0–121.0)
Hematocrit, %	33.6 (28.7–37.3)	33.8 (28.7–37.4)	33.2 (28.5–37.2)
**Iron metabolism variables**			
Iron, μmol/L	10.8 (8.0–14.0)	11.1 (8.9–14.2)	8.7 (6.8–12.3)
TIBC, μmol/L	40.8 (37.2–44.7)	40.9 (37.2–44.0)	40.1 (35.7–46.5)
**Inflammatory variables**			
Ferritin, pmol/L	994.3 (416.4–1639.4)	977.0 (409.6–1614.7)	1025.1 (593.9–1952.0)
C-reactive protein, mg/L	0.5 (0.1–1.1)	0.4 (0.1–1.1)	0.5 (0.2–1.0)
**Lipid profile**			
Cholesterol, mmol/L	4.9±1.1	5.0±1.1	4.5±1.2
Triglycerides, mmol/L	1.9 (1.5–2.7)	1.8 (1.5–2.8)	2.0 (1.5–2.6)
**Nutritional and body composition variables**			
Albumin, g/L	41.0 (39.0–43.0)	41.0 (39.0–43.0)	42.0 (40.0–44.0)
Body mass index, kg/m^2^	22.9 (20.6–26.0)	23.4±3.6	23.6±4.1
MAC, % standard	88.8±12.7	89.3±12.7	86.3±13.0
MAMC, % standard	92.5 (85.8–103.7)	92.5 (87.0–104.1)	91.3 (84.4–101.3)
CMAMA, cm^2^	38.9 (32.6–44.9)	39.4 (33.3–44.9)	35.0 (30.0–45.2)
MAFA, cm^2^	10.8 (7.1–15.3)	11.3 (7.1–16.1)	8.6 (7.1–11.2)
TSF, mm	9.6 (6.3–14.0)	10.3 (5.9–14.4)	7.8 (6.8–10.6)
Waist circumference, cm	89.6±10.0	89.6±9.9	89.6±10.5
Lean mass, kg	44.6±8.7	45.3±8.4	41.7±9.6
Fat mass, kg	13.4 (9.3–17.5)	13.7 (9.1–17.8)	11.4 (9.5–16.4)

Data are expressed as mean ± SD or median (P25—P75). Ca-P product: calcium-phosphorus product; CMAMA: corrected mid-arm muscle area; MAC: mid-arm circumference; MAFA: mid-arm fat area; MAMC: mid-arm muscle circumference; TIBC: total iron-binding capacity; TSF: triceps skinfold thicknesses.

Serum concentrations below the reference level were found for albumin in 20.2%, total cholesterol in 60.0%, and triglycerides in 41.2% of the patients. Most patients (92.1%) had serum CRP levels below 3.0 mg/L. Hypokalemia, hyperphosphatemia, and hyperkalemia were found in 17.9%, 23.5%, and 47.1% of patients, respectively. Secondary hyperparathyroidism (PTH greater than 47.7 pmol/L) was found in 31.8% of the patients.

In this study, 24.7% (n = 21) of the subjects were observed to be of underweight, 55.3% (n = 47) of normal-weight, and 20.0% (n = 17) of excess body weight by BMI. Regarding body composition, determined by WC, 22.4% and 23.5% of the patients presented high and very high risk of metabolic complications associated with obesity, respectively. By the BIA, 20% presented fat shortage and 23.5% excess fat.

To identify the predictors of mortality, Cox regression analysis was performed (Table 4). Of the three dietary fatty acid ratios evaluated (MUFA/PUFA, MUFA/SFA and PUFA/SFA), the one with a significant *p* value was used in the final model, in order to avoid collinearity. In the final model, age was an independent predictor of mortality, and one-year increase in this variable was associated with a 31.9% increase in the risk of death from all causes. Serum ferritin, calcium-phosphorus product, NO, and vitamin C intake were also positively associated with mortality. On the other hand, the increase of one unit in the dietary MUFA/PUFA ratio was associated with a 98.9% reduction in the risk of death. Lean mass, serum iron, and TSF were also negatively associated with mortality. The explanatory power of the final model was 59.3% (R^2^ = 0.469, maximum possible = 0.791), considered a good fit for survival models [38].

**Table 4:** Hazard ratios (HR)* for all-cause mortality in patients on hemodialysis at three years of follow-up.

	Crude model			Final model		
Risk factors	HR	95% CI	P-value	HR	95% CI	P-value
Age, years	1.098	1.046–1.161	**<0.001**	1.319	1.131–1.538	**<0.001**
Ferritin, pmol/L	1.001	0.999–1.002	0.279	1.001	1.001–1.002	**0.002**
Ca-P product, mmol^2^/L^2^	1.163	0.743–1.819	0.509	1.114	1.031–1.205	**0.007**
Vitamin C intake, mg	1.000	0.998–1.002	0.743	1.005	1.001–1.009	**0.007**
Nitric oxide, μmol/L	1.023	0.974–1.050	0.279	1.082	1.006–1.164	**0.034**
Lean mass, kg	0.952	0.878–1.008	0.162	0.863	0.787–0.945	**0.002**
Iron, μmol/L	0.906	0.797–1.029	0.128	0.717	0.567–0.907	**0.005**
MUFA/PUFA ratio	0.608	0.141–2.617	0.504	0.022	0.001–0.549	**0.020**
TSF, mm	0.911	0.810–1.025	0.120	0.704	0.519–0.954	**0.024**

*HR are from Cox regression models. CI: confidence interval; Ca-P product: calcium-phosphorus product; MUFA/PUFA ratio: dietary monounsaturated/polyunsaturated fatty acids ratio; TSF: triceps skinfold thicknesses.

P-values in bold present statistical significance (*p*<0.05), calculated using the Cox-proportional hazard model.

## Discussion

This is the first study, to our knowledge, to investigate the influence of vitamin C intake and dietary MUFA/PUFA ratio on the mortality of patients on HD. The dietary MUFA/PUFA ratio was negatively associated with the risk of death, that is, greater consumption of MUFA in relation to PUFA, in this population, had a beneficial effect on mortality. Dietary MUFA have cardioprotective benefits due to their anti-inflammatory action, since their consumption has been associated with lower expression of pro-inflammatory genes [39,40]. In fact, meta-analysis of cohort studies on the consumption of MUFA found positive effects on all-cause mortality and cardiovascular outcomes [41].

On the other hand, health benefits of PUFA intake vary among PUFA types. The consumption of n-3 PUFA has been related to a reduced risk of CVD and death due to its anti-inflammatory effect [42,43], while effects of n-6 PUFA intake are still controversial because of their pro- inflammatory and prothrombotic related-pathways [44]. It is worth mentioning that the result found may vary according to the fatty acid profile of the diet of each population. Therefore, evaluating and controlling the intake is important.

Still on food consumption, vitamin C intake was positively associated with mortality in this study. Although vitamin C has antioxidant properties, depending on the environment and conditions under which the molecule is active, it could also act as a pro-oxidant molecule [45]. In a study of 109 patients on HD, oral administration of vitamin C (360 and 1500 mg/week) resulted in a dose-dependent increase in plasma malondialdehyde (MDA), a compound derived from lipid oxidation and used as an oxidative stress marker [46].

We also found serum ferritin, a recognized marker of inflammation, as a positive predictor of mortality in our study. In addition to an indicator of total iron reserves, ferritin may aggravate oxidative stress and, consequently, contribute to a higher cardiovascular risk in HD patients. In studies with this population, ferritin has been considered an independent risk factor for coronary artery stenosis, especially when ≥ 600 ng/mL [47], for arterial stiffness, and peripheral arterial disease [48,49]. In addition, after vitamin C supplementation (360 and 1500 mg/week), MDA, independently correlated with serum concentrations of vitamin C and ferritin [46], suggesting that increased serum ferritin could be especially vulnerable to the pro-oxidant effects of vitamin C.

At the same time, we verified that serum iron was negatively associated with the risk of death from all causes. In fact, dialysis patients frequently receive erythropoiesis-stimulating agents and intravenous iron administration to treat anemia. However, iron homeostasis must be strictly regulated, since excess iron can act as a pro-oxidant factor, thus contributing to the oxidation of molecules, such as lipid peroxidation [50,51]. Thus, adequate monitoring of both biomarkers and careful iron administration are crucial for both adequate nutritional status and inflammation control.

Together, the findings indicate the importance of food consumption assessment, with emphasis on nutrients associated with cardiovascular risk, as well as the monitoring of inflammation biomarkers in patients on HD.

Oxidative stress evaluated by NO was also a positive predictor of mortality in our study, such that the increase of one μmol/L in serum NO concentration was associated with an increase of 8.2% in mortality. The ability of NO to preserve endothelium vasodilation depends not only on its production, but also on its rate of bioinactivation. In a reduced cell state, NO may maintain endothelial function, eliminate low concentrations of reactive oxygen species, and disrupt lipid peroxidation [52]. However, under oxidative stress conditions, NO is depleted and ONOO^-^ (peroxynitrite) accumulates, which may result in vasoconstriction, inflammation, and impairment of vascular and renal function [53]. Beberashvili et al. [54] also observed that chronic inflammation in HD patients, as measured by higher IL-6 (interleukin-6) levels, coupled with high basal NO levels seems to be associated with higher all cause and cardiovascular mortality risk.

Moreover, bone metabolism, evaluated by the calcium-phosphorus product, was another positive predictor of mortality in patients of the present study. The presence of mineral and bone disorder is common with the loss of renal function and includes all disorders of mineral and bone metabolism and their consequences, ranging from a simple alteration of serum calcium to bone diseases and fractures, and extra skeletal calcifications, mainly vascular, that are causes of the high morbimortality rate in this population [55,56].

Nutritional status and body composition were also predictors of mortality in our study, such that the increase of 1 mm in TSF and 1 kg in lean mass reduced mortality by 29.6% and 13.7%, respectively. The prevalence of protein-energy malnutrition among patients on HD is high [57,58] and, unlike the general population, epidemiological studies report an inverse association between obesity and mortality in these patients [59,60], such that a higher BMI is associated with lower mortality, known as the obesity paradox or reverse epidemiology. However, dry weight gain accompanied by a parallel increase in muscle mass is associated with greater survival [61]. Thus, it is necessary to adopt nutritional strategies that promote not only the gain of body weight, but also stimulate the increase of lean mass in these patients.

Our study has some limitations that must be considered when interpreting the results. First, the sample size was small and included subjects from a single center, which may have limited the study's power to identify significant associations other than those presented in the final model. However, we identified statistical significance in multivariate study, indicating interrelationship between variables of the study, thus our results can be considered important translation outcomes regarding study of determinants in HD mortality. Another limitation is the use of the BIA to evaluate body composition, although this instrument has been considered as a useful and complementary technique in assessing the body composition of a renal patient [62]. A third limitation would be that the level of vitamins, vitamin C and vitamin B-complex especially, in the plasma has not been determined. Lastly, FFQ requires cognitive skills of the individual to remember the consumption of food items listed during determined time, reflecting more habitual than actual diet [63]. However, this tool’s characteristic is very useful in the nutritional epidemiology [35].

In conclusion, our results suggest that dietary intake is also a predictor of mortality in HD patients, in addition to nutritional status, body composition, oxidative stress, inflammation, and bone metabolism. Thus, the evaluation of these nutritional and biochemical markers altogether can be useful in the prognosis and control of risk factors, thereby improving survival in this population. In addition, our study indicates the importance of adequate nutritional assessment, including anthropometry and food intake analyses, as well as the determination of routine biomarkers for diagnosis and control in patients, as a clinical practice in the HD service. Moreover, the use of oxidative stress and inflammation markers, in addition to the recognized markers related to uremic syndrome and comorbidities, may help in the prognosis and improve the survival of HD patients.

## Supporting information

S1 File Sociodemographic and health questionnaire, in the original language and English, used to collect information from individuals on hemodialysis. (PDF)
