# Supplementary material for: Dietary intake as a predictor for all-cause mortality in hemodialysis subjects (NUGE-HD study)
Source: PLoS One. 2019 Dec 17;14(12):e0226568. doi: 10.1371/journal.pone.0226568 (PMC6917285; doi:10.1371/journal.pone.0226568)
Supplement: S1 File — (PDF) [file pone.0226568.s001.pdf]

UNIVERSIDADE FEDERAL DE VIÇOSA

Núcleo de Pesquisa e Extensão em Nutrição Renal – NUPREN

Nutrição e Genética nos Desfechos em Hemodiálise: NUGE – HD

Questionário Sociodemográfico e de Saúde

Nº participante: ________________________

Data da coleta: _______/_______/________ Nome: ____

Data de nascimento: ______/______/_______ Idade: _________________ anos Sexo: ( ) M ( ) F

Profissão: _____________________________ Raça: ( ) Branca ( ) Negra ( ) Parda

Escolaridade: __________________________ Renda média: _________________

Data primeira sessão de HD: ____/____/____ Tempo em tratamento de HD (meses ou anos): ____________

Etiologia (causa) da doença renal: ____

Tem outro problema de saúde: ____

Apetite atual: ( ) Excelente ( ) Bom ( ) Regular ( ) Ruim

Mudanças no apetite nos últimos 6 meses: ( ) S ( ) N Causa: ____________________________________

Perda de peso nos últimos 6 meses: ( ) S ( ) N Causa: ____

Sensação de plenitude gástrica: ( ) S ( ) N Saciedade precoce: ( ) S ( ) N

Problemas de mastigação/deglutição ( ) S ( ) N Por que: ____

Adesão à dieta especial: ( ) Sim ( ) Não

Função intestinal: ____

Etilismo atual: ( ) S ( ) N Tipo bebida alcóolica/Frequência: ___________________________ Ex-etilista: ( ) S ( ) N

Fuma: ( ) S ( ) N Frequência: _________________ Ex-tabagista: ( ) S ( ) N

Faz exercícios regularmente: ( ) S ( ) N Qual (is)/Frequência: _________________________________

Medicamentos em uso: ____

Função renal residual: ( ) S ( ) N Volume urinário (L ou mL): ___________________________

Ingestão hídrica diária (mL ou L): _____________

Ganho de peso interdialítico (Média mensal): _______________________________ Peso atual:__________ Kg

Estatura: ______________________________ IMC: ______________________ Classificação:____________

UNIVERSIDADE FEDERAL DE VIÇOSA

Research and Extension Centre in Renal Nutrition

Nutrition and Genetic on Hemodialysis outcomes: NUGE – HD

Sociodemographic and Health Questionnaire

Study ID number: ______________________

Collection date:______/_______/__________ Name:____

Date of birth: ________/________/_________ Age:___________________ years Sex: ( ) M ( ) F

Occupation: ____________________________ Skin collor: ( ) White ( ) Black ( ) Brown

Escolaridade: ___________________________ Average income: _______________

Date of first HD session: _____/_____/______ Time on HD treatment (months or years): ________________

Etiology (cause) of kidney disease: ____

There is another health problem: ____

Current Appetite: ( ) Excelent ( ) Good ( ) Regular ( ) Bad

Appetite changes in the last 6 months: ( ) Y ( ) N Cause: ____

Weight loss in the last 6 months: ( ) Y ( ) N Cause: ____

Gastric fullness sensation: ( ) Y ( ) N Early Satiety: ( ) Y ( ) N

Chewing/Swallowing Problems: ( )Y ( ) N Why: ____

Adherence to special diet: ( ) Yes ( ) No

Intestinal Function: ____

Current Ethylism: ( ) Y ( ) N Alcohol Type/Frequency: ___________________________ Former alcoholist: ( ) Y ( ) N

Smoke: ( ) Y ( ) N Frequency: __________________ Former smoker ( ) Y ( ) N

Do exercises regularly: ( ) Y ( ) N What/Frequency: ____

Medicines in use: ____

Residual renal function: ( ) Y ( ) N Urinary Volume (L or mL): _________________________________

Daily water intake (mL or L): ________________

Interdialytic Weight Gain (Monthly Average): ______________________________ Current Weight:__________ Kg

Height: _______________________________ BMI: _______________________ Classificação:_________
